# Use of Osteopathic Manipulative Treatment for the Management of Pectus Excavatum: A Single Case Study

**DOI:** 10.7759/cureus.61005

**Published:** 2024-05-24

**Authors:** Zhao Xiang Lin, Anna Perez, Mikhail Volokitin

**Affiliations:** 1 Osteopathic Manipulative Medicine, Touro College of Osteopathic Medicine, New York City, USA

**Keywords:** sternal manipulation, chest wall compression, dyspnea on exertion, osteopathic manipulative treatment, pectus excavatum

## Abstract

Pectus excavatum (PE) is a congenital defect that presents with an anterior depression of the chest wall, which can impact cardiopulmonary function. A 25-year-old hypermobile male presented with a history of PE and chronic dyspnea on exertion, chronic cough, and intermittent chest wall pain. This study explores osteopathic manipulative treatment (OMT) as a possible alternative to improve symptoms associated with PE. Osteopathic structural exam (OSE), volumetric measurements of the thoracic cavity, vitals, and pulmonary function tests were evaluated at baseline and after OMT. The patient was treated with 14 weeks of weekly OMT for his exertional dyspnea, cough, and chest wall pain. Somatic dysfunctions were addressed through OMT, which all improved by the end of the 14-week treatment. Notably, the excursion at the sternal angle increased by threefold after complete treatment. The patient reported subjective improvement in all symptoms, with durable improvement in chest wall pain at 10 months after cessation of treatment. The application of OMT can help alleviate symptoms of pectus excavatum and aid in the management of patients who have not received surgical interventions.

## Introduction

Pectus excavatum (PE) is a congenital defect that presents with an anterior depression of the chest wall. It is the most common congenital chest wall defect, and it manifests more commonly in men, with a 3:1 male-to-female ratio [1, 2]. The presentation of PE can be linked to familial inheritance, with a prevalence of approximately one out of every 400 births. Studies have also shown that PE often presents in conjunction with other heritable disorders of connective tissue and in hypermobile individuals [3]. Symptoms range from general pain and discomfort to exercise intolerance and tachycardia and can impact the function of the heart and lungs, depending on the severity of PE.

Patients who do not present with severe PE can be asymptomatic at rest but can often experience exercise intolerance [4, 5]. This limitation in physical exercise has been suggested to be a cardiovascular performance issue due to the compression of the anterior chest wall inhibiting cardiac output. Additionally, PE patients can also present with rib flare, which is thought to be secondary to anomalies in the diaphragm producing posterior traction on the sternum [6]. Currently, only surgical correction has been shown to significantly improve cardiopulmonary function at rest and during exercise [7]. Physical therapy and vacuum bells can be prescribed to help with the management of PE [8]. Our study hypothesizes that osteopathic manipulative treatment (OMT), which focuses on the musculoskeletal system, can help address chest wall structural deformities in PE [9, 10]. In this study, we aim to examine the use of OMT to address somatic dysfunctions (SD) of the rib, thorax, and diaphragm to improve management and other PE-associated symptoms in a 25-year-old male with PE.

## Case presentation

**Figure 1:**
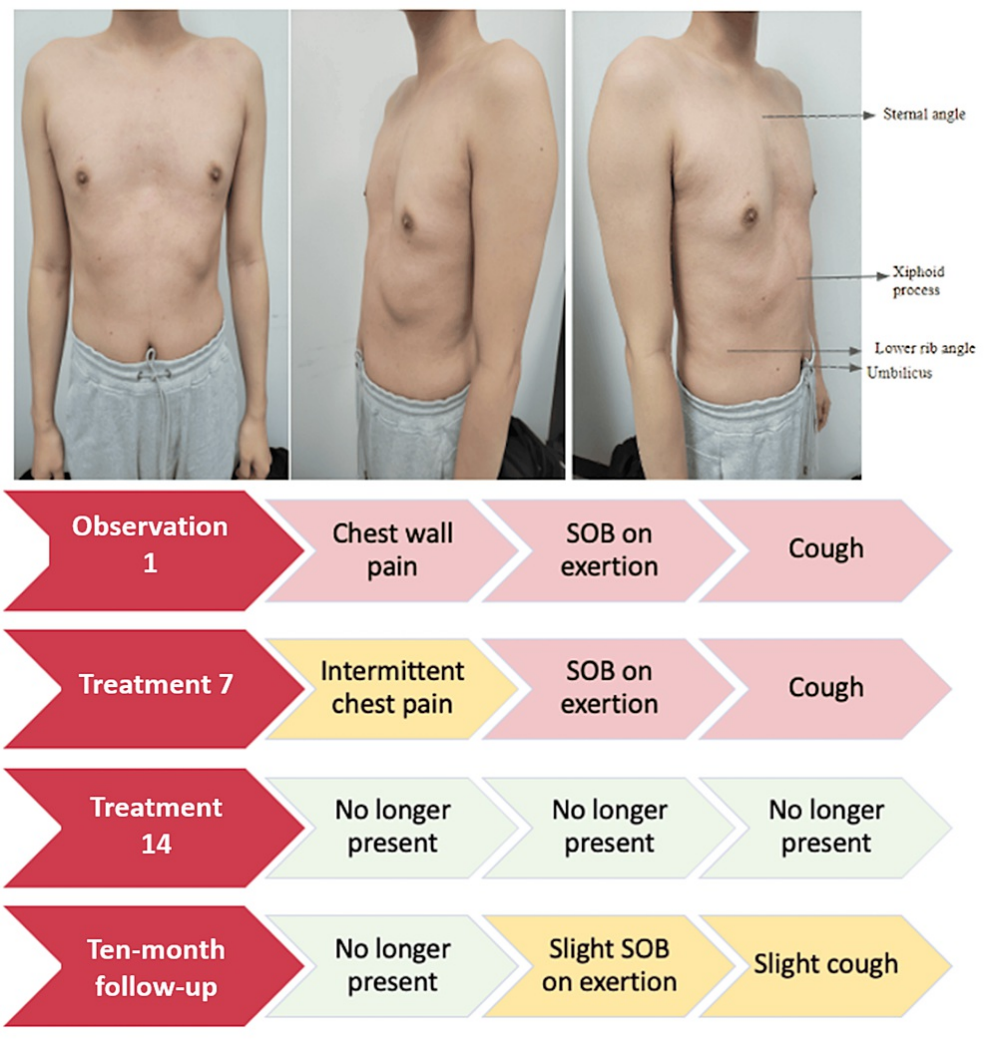
Patient presentation with pectus excavatum and treatment results SOB: shortness of breath

A 25-year-old hypermobile male presented with a history of PE and chronic dyspnea on exertion, a chronic cough, and intermittent chest wall pain. He reported a history of “weak lungs” as a child, which paradoxically improved following a swine flu (H1N1) influenza infection. The initial presentation is shown in Figure 1. The patient was instructed to practice stretching and breathing exercises daily in conjunction with weekly OMT, for a total of 14 treatments. 

The patient’s resting heart rate, respiratory rate, blood pressure, and oxygen saturation were measured prior to the application of OMT and re-measured each week until the completion of the 14-week treatment course. Anthropometric and volumetric measurements included the diameter and width of the chest, which were measured with a ruler and tape measure to determine the severity of PE. The sternum, rib angle, and degree of rib flare will also be measured before and after the use of OMT. A spirometry test was used to assess pulmonary function. Forced expiratory volume in the first second (FEV1) and forced vital capacity (FVC) were obtained from the patient before each OMT session and used to calculate the FEV1/FVC ratio.

An osteopathic structural exam (OSE) was conducted by an osteopathic medical student under the supervision of an osteopathic manipulative medicine (OMM) department faculty member at Touro College of Osteopathic Medicine each week during the 14-week duration prior to the use of OMT. The emphasis was placed on examining the rib cage, sternum, xiphoid, thoracic spine, and inlet, as well as the diaphragm. Other areas were not examined. To better gauge the movement of the diaphragm, the patient was prone, and the diaphragm was palpated anteriorly and posterolaterally from the thoracic cage. The OSE was aimed at determining the presence of rib, thoracic, and/or diaphragmatic SD, as well as inhalation and exhalation SD for pump and/or bucket motion of the ribs. Costovertebral articulations were evaluated for a possible increase in sympathetic tone, given the anatomic proximity of the sympathetic chain ganglia immediately anterior to the costovertebral articulations. Atlanto-occipital junction (OA) and occipitomastoid suture (OM) restrictions were evaluated to assess parasympathetic tone. C3-C5 of the cervical area was also assessed to address issues involving the phrenic nerve.

The OMT techniques used included seated rib raising, seated rib articulation, myofascial release of erector spinae muscles, muscle energy of thoracic spine SDs and rib inhalation and exhalation SDs, balanced ligamentous tension (BLT) of the sternum and 12th ribs, facilitated positional release (FPR) of the clavicle and first rib, doming of the diaphragm, OA, and OM release. These interventions were done at every treatment, but specific dysfunctions treated were adjusted based on the dysfunctions found at each visit. The order of interventions was in part determined to address patient comfort in positioning, starting from a seated position before transitioning to a supine position. The goal of starting each session with a seated rib raise was to normalize sympathetic tone in preparation for the rest of the techniques. From here, we continued with passive techniques, namely articulation and myofascial release, and progressed to active techniques, specifically muscle energy. Once the patient was transitioned to a supine position and active techniques were completed, any remaining ligamentous strains following the release of muscle hypertonicity were addressed with BLT and FPR. The treatment ended with techniques focused on normalizing parasympathetic tone to balance overall autonomic tone. The results of the OSE are demonstrated in Table 1. Patient response after treatment #14 is shown in Figure 1. 

**Table 1:** Osteopathic structural exam (OSE) and maximal excursion results

OSE	Baseline	Treatment #7	Treatment #14
Thoracic spine	T1-8NSrRl with flattening of kyphosis at those levels, T10-L3NSlRr, sway back posture	T1-8NSrRl, subtle with flattening of kyphosis at those levels, sway back posture	Sway-back posture
Sternum	Concavity of the sternum from rib 6 to the xiphoid process at the midline with right side-bending restriction in the frontal plane and vertical axis rotation restricted to the left	The concavity of the sternum from rib 6 to the xiphoid process at midline, frontal plane, and side-bending to the right in the upper half is restricted; vertical axis rotation is restricted on the right.	Concavity of the sternum from rib 6 to the xiphoid process at the midline
Ribs	Left thoracic cage rotation, exhalation dysfunction of ribs 1–5 on the left, and 12th ribs bilaterally	Left thoracic cage rotation, low ribs laterally restricted to the left, and inhalation restriction of ribs 2-4 on the left	Limited motion of the first rib on the right; inhalation dysfunction of rib 3 on the left; exhalation dysfunction of rib 5 on the right
Diaphragm	Bilaterally inhaled hemidiaphragms	Inhalation dysfunction on the left	No dysfunction was noted.
Maximal excursion (cm)	Diaphragm: 4; sternal angle: 2; xiphoid process: 5; lower rib angle: 4; umbilicus: 2	Diaphragm: 4; sternal angle: 5; xiphoid process: 5; lower rib angle: 4; umbilicus: 2	Diaphragm: 5; sternal angle: 6; xiphoid process: 6; lower rib angle: 4; umbilicus: 4

The pulmonary function was measured with a spirometer, and results show an increase in FVC from 86.8% predicted at baseline to 114.2% predicted at treatment #7. Similarly, FEV1 also shows an increase from 89.9% predicted at baseline to 117.5% at treatment #7. The FEV1/FVC ratio has remained consistent, from an initial 87.20% at baseline to 87.80% at treatment #7. Subsequently, the final results at treatment #14 remained relatively unchanged from treatment #7. The respiratory rate decreased by over 33% from 18 breaths per minute at baseline to 12 breaths per minute at treatment #14. The chest wall and abdominal maximal excursion are shown in Table 1. The chest wall maximal excursion increased by 4 cm to 5 cm for the diaphragm, increased by 2 cm to 6 cm for the sternal angle, and increased by 5 cm to 6 cm for the xiphoid process from baseline to treatment #14. The abdominal wall maximal excursion increased by 2 cm to 4 cm for the umbilicus, while the lower rib angle remained unchanged from baseline to treatment #14.

## Discussion

The purpose of this case study was to present the effects of an integrated OMT intervention for an individual with pectus excavatum. The patient tolerated the prescribed interventions very well. The positive results of increased pulmonary function and maximal recorded excursions improved the patient’s symptoms of chest pain, cough, and dyspnea on exertion and overall quality of life.

The patient initially noted some improvement in chest wall pain at treatment #7. At this time, it was also noted that there was an improvement in kyphosis of the thoracic spine, increased motion at the right hemidiaphragm, excursion at the sternal angle, pulse, and respiratory rate. The improvement in chest wall pain may be in part due to increased mobility of the chest wall, as noted by increased excursion at the sternal angle and improved kyphosis of the thoracic spine [11, 12]. Inhalation dysfunction of the diaphragm (hypertonicity of the diaphragm resulting in flattening) has been associated with paradoxical rib retractions with inhalation (Hoover sign), which may further explain the resolution of exhalation dysfunctions in the left one to five ribs and bilateral 12th ribs and improvement of motion in the right hemidiaphragm between the baseline evaluation and treatment #7 [13]. These findings are associated with improvements in pulmonary function tests between baseline and treatment #7, despite the patient continuing to report exertional dyspnea and cough.

By treatment #14, the patient reported improvement in chest wall pain, exertional dyspnea, and cough. Additionally, the thoracic spine kyphosis and type I group curve resolved, and both sternal and diaphragmatic mobility significantly improved. New inhalation and exhalation dysfunctions of the ribs were present at the time of treatment #14, without a decline in pulmonary function testing. Symptomatic improvement was not significantly correlated to rib inhalation or exhalation dysfunction but was rather associated with improvement in the resolution of the constellation presentation of left thoracic rotation, dextroscoliosis, flattening of the thoracic kyphosis, and flattening of the left hemidiaphragm. As the thoracic cavity is comprised of the thoracic spine, ribs, and sternum, improved sternal mobility is likely related to an overall improvement in chest wall mobility [14]. Rib-raising techniques beneficially affect the sympathetic innervation at the chain ganglia by articulating the rib heads by lifting and rotating them through their fascia attachments. For this technique, gentle anterior pressure is applied to the ganglia that lie anterior to their corresponding ribs as the patient lies supine. This produces a short-lived initial increase in sympathetic activity but is followed by a long-lasting sympathetic inhibitory effect. Rib raising has also been shown to improve the negative thoracic pressure for maximum inhalation and improve breathing. According to a pilot study, sympathetic nervous system activity may decrease after rib raising [15]. Myofascial release is a treatment technique that engages continual palpatory feedback to achieve the release of myofascial tissues, improving pain, decreasing inflammation, and potentially improving wound healing. Balanced ligamentous tension was used to address ligamentous articular strains. Muscle energy treatment was applied to resolve the reflex, maintaining corresponding somatic dysfunction via reciprocal and autogenic inhibition [16].

Proposed mechanisms of the pathogenesis of PE include intrauterine pressure or diaphragm dysfunction resulting in posterior traction on the sternum, which then compresses the mediastinum [6]. Studies have shown that the limitation in physical exercise for PE patients likely stems from a cardiovascular performance issue due to the compression of the anterior chest wall inhibiting cardiac output rather than a decrease in lung capacity [6, 17]. This can be seen by the limited enhancement in pulmonary function testing of the patient during and after OMT, despite the improvement of symptoms reported for dyspnea on exertion, chest wall pain, and cough. In contrast, looking at the three-fold increase in excursion at the sternal angle and considering the anatomic relationship between the sternum and the heart, improvements in symptoms suggest that OMT can reduce anterior chest wall compression and musculoskeletal SD of the thoracic cage. The improvement in symptoms, physical exam, and OSE findings in this patient with PE underscore the potential utility of OMT as a potential adjunct therapy alongside surgical correction or as an early intervention in adolescent patients whose sternums have not yet begun to fuse [18].

There are several limitations in this case report. Conducting a cardiac stress test could have provided more insights into the effects of OMT on cardiovascular function [19]. Consideration was also given to the patient’s financial status as to whether imaging and stress testing would be ordered. Due to the patient’s limited financial means, additional imaging and testing were not obtained. Lastly, this study focuses on the use of OMT on a single PE patient. Case-series, case-control, or cohort studies would be valuable in ascertaining whether using OMT as a management strategy provides benefits to a wider range of patients, including younger patients with more malleable sternums.

## Conclusions

By prescribing a 14-course weekly treatment plan, the patient’s reported symptoms resolved with structural improvements leading to increased pulmonary function and quality of patient’s life. Improvement in chest pain endured even at a 10-month follow-up post-treatment, while there was a return of symptoms at a decreased severity for cough and dyspnea on excursion. This suggests the benefit of continued treatment after 14 weeks in the management of PE with OMT. With further investigation into a larger patient population, OMT application can be more commonly utilized in the management of patients who have not received surgical interventions.
